# Eddy Current-Based Delamination Imaging in CFRP Using Erosion and Thresholding Approaches

**DOI:** 10.3390/s24185932

**Published:** 2024-09-13

**Authors:** Dario J. Pasadas, Mohsen Barzegar, Artur L. Ribeiro, Helena G. Ramos

**Affiliations:** Instituto de Telecomunicações, Instituto Superior Técnico, Universidade de Lisboa, 1049-001 Lisbon, Portugal

**Keywords:** eddy current testing, delamination, CFRP, damage imaging, non-destructive evaluation

## Abstract

Carbon fiber reinforced plastic (CFRP) is a composite material known for its high strength-to-weight ratio, stiffness, and corrosion and fatigue resistance, making it suitable for its use in structural components. However, CFRP can be subject to various types of damage, such as delamination, matrix cracking, or fiber breakage, requiring nondestructive evaluation to ensure structural integrity. In this context, damage imaging algorithms are important for assessing the condition of this material. This paper presents signal and image processing methods for delamination characterization of thin CFRP plates using eddy current testing (ECT). The measurement system included an inductive ECT probe with three coil elements, which has the characteristic of allowing eddy currents to be induced in the specimen with two different configurations. In this study, the peak amplitude of the induced voltage in the receiver element and the phase shift between the excitation and receiver signals were considered as damage-sensitive features. Using the ECT probe, C-scans were performed in the vicinity of delamination defects of different sizes. The dimensions and shape of the ECT probe were considered by applying the erosion method in the damage imaging process. Different thresholding approaches were also investigated to extract the size of the defective areas. To evaluate the impact of this application, a comparison is made between the results obtained before and after thresholding using histogram analysis. The evaluation of damage imaging for three different delamination sizes is presented for quantitative analysis.

## 1. Introduction

Composite materials such as carbon fiber reinforced polymers (CFRPs) are widely used in aerospace, automotive, and structural applications due to their high strength-to-weight ratio and excellent fatigue resistance [1]. Delaminations are a critical form of damage in these composite materials that can propagate under loading conditions and lead to catastrophic failure. Therefore, their detection and characterization are essential to ensure the structural integrity and longevity of the CFRP structures [2,3]. Their detection is challenging with traditional visual inspection techniques as delaminations occur between layers of composites and are not usually visible from external surfaces. Therefore, different types of nondestructive testing (NDT) techniques have been proposed for the detection of delaminations in composite materials, including acoustic emission testing [4], ultrasonic testing [5], guided wave testing [6], infrared thermography testing [7], and eddy current testing (ECT) [8]. Most of these techniques require contact measurements. ECT is a valuable technique for inspecting conductive materials and detecting surface and near-surface defects without physical contact between the probe and the specimen. Among non-contact methods, ECT is less complex and less expensive with high effectiveness in various environmental conditions. This technique works with the principle of electromagnetic induction [9]. A time-varying current flowing through a coil produces a primary magnetic field, and if the coil is close to a conductive material, this primary magnetic field induces eddy currents in the specimen. These induced currents produce a secondary magnetic field. Discontinuities, such as fiber breaks or delamination, disturb the flow of eddy currents and cause detectable changes in the secondary magnetic field [10,11]. Magnetic field sensors are typically used to measure these variations. By analyzing these magnetic field variations, it is possible to detect and characterize defects in the specimen. In CFRPs, due to the low electrical conductivity of the material, the sensitivity of an ECT probe is low, which is challenging for damage detection. Several studies have focused on improving the sensitivity and accuracy of ECT for damage detection in CFRPs. Advances in sensor technology, including the development of high-frequency ECT probes and multi-coil arrays, have significantly enhanced the detection of defects in composites. For example, D. Wu et al. [12] proposed a highly sensitive ECT probe with an 8-shape to detect cracks in CFRPs. The results show that by using their probe, the influence of the lift-off variations during the scanning process was reduced even at high testing frequencies of 10 MHz to 25 MHz. D. Pasadas et al. [13] proposed an ECT probe with the use of resonant excitation/sensing circuits and double excitation coils to enhance defect detection in CFRPs. The results show that the use of resonant frequency techniques in both excitation and sensing circuits can significantly improve the detectability of cracks in CFRP materials. M. Machado et al. [14] focused on high-speed inspection of CFRP materials for delamination detection using eddy current testing.

In the context of ECT, the extraction of appropriate features and parameters that can analyze and determine the defects presence, location, and characteristics is essential. Perturbations in the amplitude and phase in the detected signals indicate the presence of damage in the structure [15,16]. Variations in the impedance of the ECT coils can also indicate the presence of defects [17,18]. The analysis of the response of the ECT probes at different frequencies can also assist in the evaluation of the depth, location, and type of defects [19,20]. Parameters such as the signal-to-noise ratio of the resulting ECT images can be also analyzed for damage assessment [21]. Nevertheless, efficient damage characterization requires appropriate signal processing techniques to interpret ECT data.

This study explores the use of a high-frequency ECT probe and signal and image processing methods for delamination assessment of CFRPs. Due to the diffusion of eddy currents in the CFRP plate and the spatial resolution of the ECT probe, accurate damage imaging and sizing can face some challenges. From the resulting images obtained by the ECT probe, a morphological operation was applied to refine the detected defect boundaries considering the shape of the ECT probe. Thresholding approaches using Otsu’s and global thresholding methods were implemented to improve the accuracy of the represented image of the defective region. The automatic thresholding approach of Otsu’s method selects a threshold by maximizing the between-class variances of pixel intensities between a defective region and the background. This automatic approach was compared with two global thresholds (−3 db and −6 dB). The evaluation of damage imaging for different defect sizes is presented for quantitative analysis.

The paper is organized into seven sections. After this introduction, Section 2 introduces the experimental setup used in this study, which includes three different CFRP specimens with different delaminations, as well as the ECT probe characteristics used for the inspection. Section 3 provides detailed descriptions of the extracted features and the methodology used for imaging and estimating delamination sizes. Section 4 presents the C-scan images obtained by the ECT probe in different excitation conditions and for different delamination sizes. Section 5 and Section 6 describe the erosion and thresholding approaches, respectively, used in the implemented algorithm to assist in imaging and estimating delamination size. The results of each implementation are presented in the referred two sections, and quantitative analyses of the estimated defect parameters based on the imaging results are described in Section 7. The conclusions are provided in Section 8.

## 2. ECT Inspection Process and Setup

This section provides a detailed description of the material, experimental setup, and procedures used to detect and assess delaminations in the CFRP specimens. In Section 2.1, a description of the CFRP specimens under test is presented. In Section 2.2, details of the ECT probe are presented. In Section 2.3 the measurement system and the experimental procedures used to perform the inspection of each CFRP plate are presented.

### 2.1. Characteristics of the Specimens under Test

**Figure 1:**
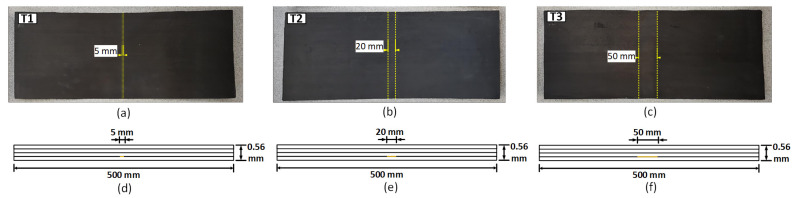
Representation of the three specimens under test with delaminations of different lengths from the top view: (**a**) 5 mm; (**b**) 20 mm; (**c**) 50 mm; and the corresponding profile view of the mentioned delaminations: (**d**) 5 mm; (**e**) 20 mm; (**f**) 50 mm.

Three CFRP plates, denoted as T1, T2, and T3, each with dimensions 500 × 200 × 0.56 mm^3^, were considered for the study. Delaminations were introduced in the specimens by inserting Teflon films between the fiber layers. The plates were fabricated using pre-impregnated composite fibers. Each CFRP plate consisted of four fiber layers oriented in the sequence of [0/90/90/0]. Figure 1 provides the schematic profile of the plates and photographs of the CFRP plates. The delaminations covered the entire width of the plates and extended 5 mm, 20 mm, and 50 mm in the length direction, as shown in Figure 1a–c, respectively. A schematic of the profile view of the CFRP plates is presented in Figure 1d–f, showing extensions of 5 mm, 20 mm, and 50 mm in the length direction, respectively.

### 2.2. ECT Probe

**Figure 2:**
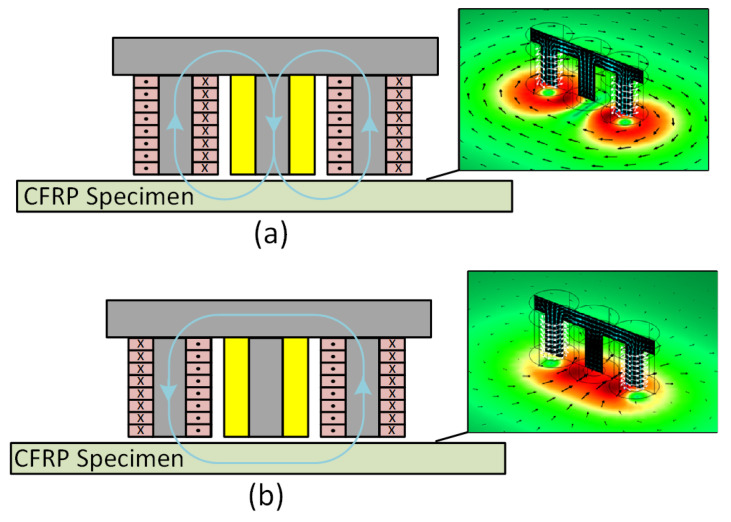
Characteristics of the ECT probe used to inspect the CFRP specimens based on (**a**) excitation configuration 1; (**b**) excitation configuration 2.

The type of probe and the selection of testing frequencies are important for obtaining high-quality scanned images. Given that CFRP structures exhibit low electrical conductivity, they require ECT probes operating at high frequencies for effective inspection. Using an inductive coil as a sensing element at higher frequencies enhances sensitivity and resolution, improving the detection of small variations in material properties and thus enhancing the accuracy of defect characterization. In this study, an ECT probe with double excitation coils and one sensing coil were used to obtain C-scan images of the delamination regions. Figure 2 shows a schematic of the ECT probe. The probe has 50 turns for each excitation coil and 160 turns for the sensing coil, thereby increasing the detector’s sensitivity. The external diameter of each coil is approximately 4.5 mm, and their heights are equal to 4 mm. The total size of the probe is 13.5 mm × 4.5 mm × 6 mm (length × width × height). The sensing coil was strategically positioned between the two excitation coils to measure the induced voltage (V) generated in the center of the probe. This induced voltage is proportional to the time derivative of the magnetic flux linked to the sensing coil. This probe, which has been used for detecting fiber breaks [10], can be used to evaluate the material’s condition using two different excitation configurations. These configurations are achieved by connecting the two excitation coils either in series with the same wire direction or with opposite wire directions. When the two excitation coils are connected in series with the same wire direction, the excitation current causes eddy currents to flow around the sensing coil and the magnetic field to circulate along the central ferrite core. In this paper, this excitation configuration is referred to as configuration 1, as shown in Figure 2a. When the two excitation coils are connected in series with opposite wire directions, the excitation current causes eddy currents to flow below the sensing coil. This excitation configuration is referred to as configuration 2, as shown in Figure 2b. Ferrite cores are used to concentrate the magnetic field in the center of each coil. For each configuration, the orientation of the magnetic field is depicted in blue lines. Both configurations were used in this study to evaluate their damage detectability in the case of delamination.

### 2.3. Experimental Setup and Procedures Used for the ECT Inspection

**Figure 3:**
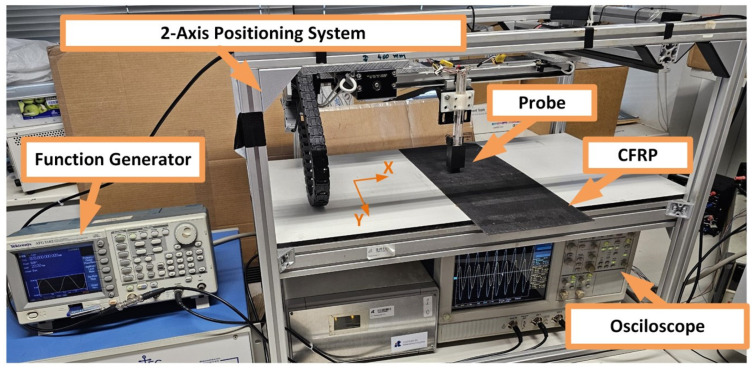
Experimental setup.

**Figure 4:**
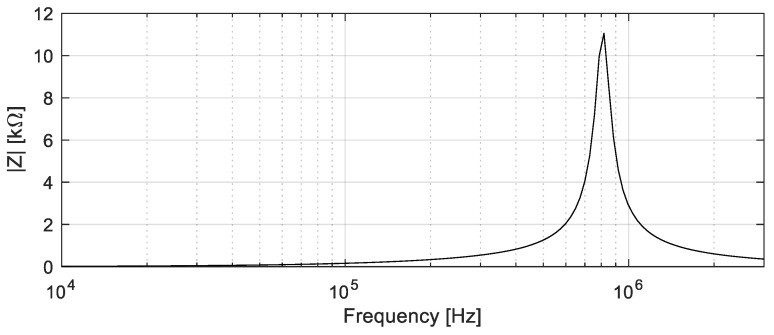
Impedance-frequency characteristic curve of the sensing element of the ECT probe.

The measurement system used to evaluate the condition of the CFRP plates is presented in Figure 3. The ECT probe was attached to a two-axis positioning system to perform a C-scan around the location of each delamination. The probe was fixed to the scanning system with a lift-off of 0.5 mm between the surface of the CFRP specimen and the bottom of the probe. Given that that CFRP structures have low electrical conductivity, it requires ECT probes to operate at high testing frequencies to inspect these materials. The testing frequency was chosen to operate at a frequency within the near electrical resonance band to enhance the sensitivity and signal-to-noise ratio of the ECT probe [22]. The impedance characteristic curve of the sensing element, which is shown in Figure 4, was measured using an LCR meter (3532-50 from Hioki, Nagano, Japan), considering the capacitance of the coaxial cable and the capacitance of the oscilloscope channel. Accordingly, a sinusoidal signal of 0.81 MHz of testing frequency with a peak-to-peak amplitude of 20 V was applied to the excitation coils using a function generator (AFG3102 from Tektronix, Beaverton, OR, USA). The use of an inductive coil as a sensing element at higher testing frequencies provides improved sensitivity and resolution for detecting small variations in the material properties, enhancing the accuracy of the defect characterization. However, at this frequency, the background noise, due to the surface conditions and the lift-off variations, can be also detected. Therefore, for each position of the probe, a signal averaging of 30 measurements was performed to increase the signal-to-noise ratio. The scans were performed with the three coils aligned with the y-axis of the positioning system. The positioning system moved the probe in steps of 1 mm over a region of 130 × 10 mm^2^ around the position of each delamination. The measurement system was maintained with stable connections to minimize the risk of parasitic capacitances that can appear in the excitation and sensing circuits. An oscilloscope (TDS5032B from Tektronix, Beaverton, OR, USA) was used to acquire the voltage across the excitation coils and sensing element. A personal computer was used to collect these data through a GPIB interface and control the positioning system through the RS232 interface. The results obtained for each scan are presented in Section 4.

## 3. Methodology for Imaging and Estimating Delamination Size

**Figure 5:**
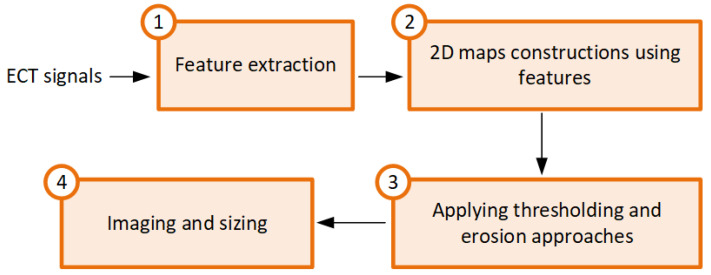
Workflow of the methodology used for imaging and sizing of the delaminations in the CFRP plates.

A workflow chart depicting the signal and image processing stages applied to the direct ECT images to characterize the delaminations is depicted in Figure 5. Features were extracted from the ECT probe to evaluate the conditions of the CFRP specimens. As features, the amplitude of the voltage across the sensing element and the phase shift between the sensing coil and the excitation source were extracted. From the obtained 2D maps, two image processing techniques were implemented to enhance the images of the defective region. One approach was used to adjust the 2D images of a feature considering the dimensions of the ECT probe using erosion operation. This procedure helps to reduce the effect of probe size, providing more accurate information about the defect size. Also, thresholding approaches were employed to identify and isolate regions of interest (defective regions). The two implemented approaches are described in detail in the following sections.

## 4. C-Scan Images of the ECT Data

**Figure 6:**
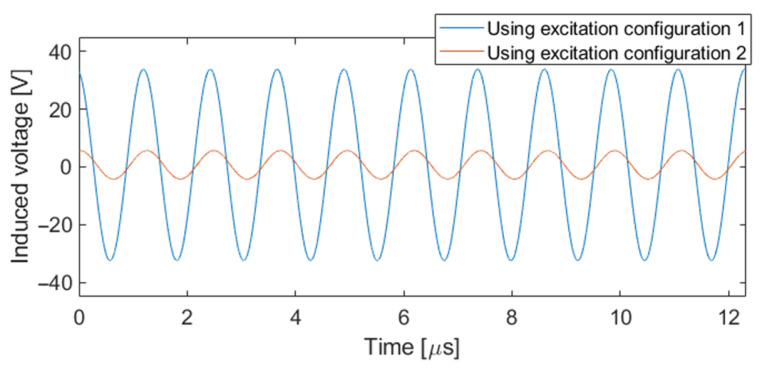
Signals of the sensing element of the ECT probe applying the two different excitation configurations.

As mentioned in Section 2.2, the ECT probe has the characteristic of allowing two different excitation configurations to evaluate the condition of the material by connecting the two excitation coils in series with same or opposite wire directions. Figure 6 illustrates an example of the original signals obtained with the oscilloscope from the sensing element of the ECT probe for each of the excitation configurations when the probe is placed in the proximity of the sample.

**Figure 7:**
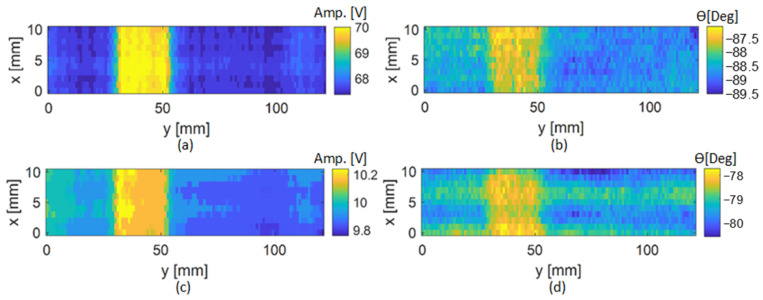
Representation of the C-scans performed in the vicinity of the 20 mm delamination: (**a**) amplitude map obtained using the excitation configuration 1; (**b**) phase shift map obtained using excitation configuration 1; (**c**) amplitude map obtained using excitation configuration 2; (**d**) phase shift map obtained using excitation configuration 2.

In this experimental study, particular attention was paid to compare the results obtained with the two excitation configurations by analyzing the perturbed amplitude of the induced voltage in the sensing element and the phase shift between the excitation and reception signals. The original scan data, which are directly acquired using the oscilloscope, include the peak-to-peak amplitude and phase shift between the excitation and receiver signals. The C-scans were performed with the delaminations located at 0.42 mm below the surface of the CFRP plates, i.e., between layers 3 and 4. The resulting C-scan maps of the amplitude and phase shift will be referred to as Amp and ϴ, respectively. Figure 7 shows an example of the amplitude and phase shift maps obtained using the two different excitation configurations for the test sample T2, where the amplitude maps are represented in volts and the phase shift maps are represented in degrees. Figure 7a,b show the results obtained using configuration 1, and Figure 7c,d show the results obtained using configuration 2.

**Figure 8:**
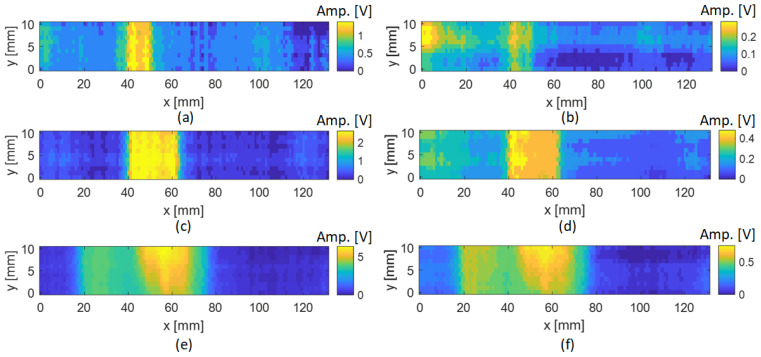
C-scan representations showing the maximum amplitude variation for different delaminations and excitation configurations: (**a**) 5 mm delamination using excitation configuration 1; (**b**) 5 mm delamination using excitation configuration 2; (**c**) 20 mm delamination using excitation configuration 1; (**d**) 20 mm delamination using excitation configuration 2; (**e**) 50 mm delamination using excitation configuration 1; (**f**) 50 mm delamination using excitation configuration 2.

From the results, it is observed that the presence of a delamination in the inspected region produces a variation in the amplitude and phase shift in both excitation configurations. These variations occur due to the presence of the defect in the material that perturbs the eddy currents from their normal path. For excitation configuration 1, when the probe was positioned away from the delamination region, the sensing element showed a peak-to-peak amplitude of approximately 67.5 V. This is because, in configuration 1, the magnetic flux is concentrated in the central ferrite core of the sensing element along the z-axis, making the sensing element more sensitive to the strong magnetic field produced in that direction. However, in configuration 2, the obtained amplitude was significantly lower due to a different distribution of the magnetic flux. Based on the level of noise observed in the phase shift maps, only the amplitude maps were considered for damage imaging. Figure 8a,c,e depict the amplitude maps obtained for the inspection of the three delaminations using configuration 1. Figure 8b,d,f depict the amplitudes maps obtained for the inspection of the three delaminations using configuration 2. The results presented in Figure 8 show that, using configuration 1, where the two excitation coils are connected in the same direction, the delaminations are clearly visible in the ECT images, and the amplitude change is significant enough to identify the three defects. However, using excitation configuration 2, where the two excitation coils are connected in opposite directions, the delaminations are still visible in the ECT images, but the amplitude change is considerably lower than that in the first configuration. Thus, the first configuration is more sensitive to the variation in the magnetic field in the presence of delamination in the CFRP specimen. It is also possible to observe that the area of amplitude variation obtained in the ECT images is larger than the real delamination size. This is due to the diffusion of the eddy currents into the plate and the spatial resolution of the ECT probe. Considering the dimension of the sensing element, the erosion method was employed to adjust the images of the amplitude features for better accuracy of the delamination size estimation.

## 5. Erosion Method for ECT Data

In ECT, the spatial resolution and accuracy depend significantly on the size of the probe. The erosion method is an image processing technique typically employed to reduce the size of objects and remove noise in the resulting image. In this study, the erosion method was employed for the correction of the obtained ECT images, taking into consideration the size of the ECT probe. This process involved using a structuring element that corresponds to the sensing element size to reduce the boundaries of the detected variations in the feature images. In this case, the shape and size of the structuring element is a circle with a radius of 3 mm considering the outer radius of the receiver coil. The value of each point in the output image is determined by the minimum value of the points in the neighborhood defined by the structuring element.

In this paper, the structuring element is defined as *p*, and the 2D map containing the variation of each feature is defined as *M*. Hence, the erosion operation is defined by the following equation:

(1) $$I(x,y)=\underset{\left(i,j\right)\in P}{\min}\{M\left(x+i,\;y+j\right)-p(i,j)\}\;\;$$

where (*x,y*) are the coordinates of the 2D map of feature *M*, (*i*,*j*) is the coordinate of *p* in the entire domain of the structuring element *P*, and *I* is the output image. 

**Figure 9:**
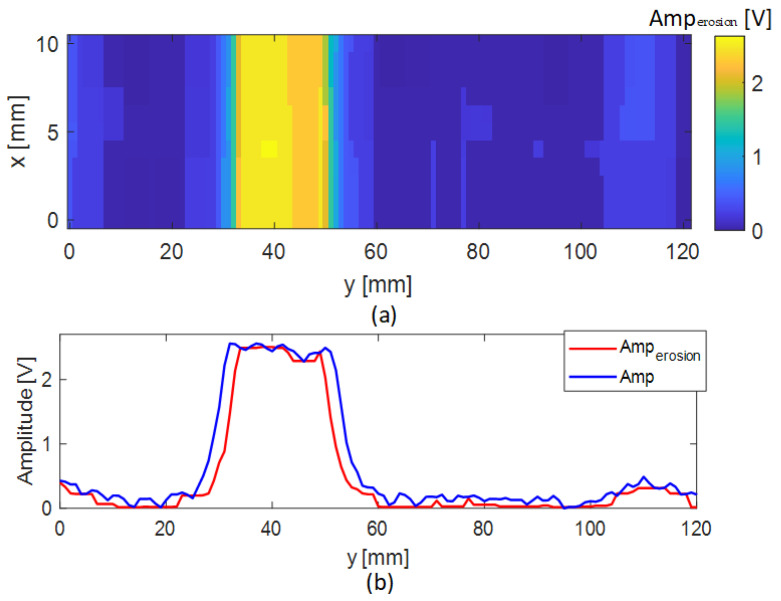
Results of applying the erosion method on an amplitude map: (**a**) C-scan image after applying the erosion method; (**b**) *Y*-axis view of the results before and after applying the erosion method.

In this paper, the resulting image of the amplitude map after employing the erosion method will be referred to as Amp_erosion_. For example, Figure 9a depicts the results of the Amp_erosion_ map for the case of Figure 8c. Figure 9b depicts an average of the y-line plots before and after applying the erosion procedure.

From Figure 9, it is possible to notice that a reduction of the region of the perturbation along the *y*-axis is visible in the results. This image processing procedure was repeated for the amplitude maps shown in Figure 8a,c,e. In Section 7, a comparison of the accuracy of the delamination length estimation before and after applying the erosion method is presented for all cases.

## 6. Thresholding Approaches for Damage Evaluation

In ECT imaging, thresholding approaches are important to extract regions of interest, such as defective areas or material irregularities for detailed analysis. In this study, three thresholds were considered and implemented to extract an image of the defective area. The first two thresholds are −3 dB and −6 dB, which refer to the global thresholding values that were used to identify significant changes in the ECT images. The third approach was using Otsu’s method, which is an automatic threshold-based segmentation approach. In all the approaches, *M* was normalized in the range of [0, 1], denoted as $\bar{M}$, before applying the thresholds. To implement the first two threshold approaches, the data were represented using $20\log_{10}\bar{M}$. A −3 dB drop in amplitude represents 70.7% of its original value, while a −6 dB drop in amplitude represents 50% of its original value. For the last thresholding method, the Otsu’s method was used to automatically extract the details of the defective areas in the ECT images. The basic idea of this method is to find an optimal threshold that separates the pixels into two classes: the target (defective areas) and the background. This is achieved by minimizing the interclass weighted variance between the classes, which effectively maximizes the variance between the target and the background. The following sequence of steps is involved in this approach: (a) computation of the histogram of the grayscale image, (b) normalization of the frequency of each intensity level, and (c) computation of the cumulative probability distribution function and the cumulative mean. After these calculations, the between-class variance $s_{B}^{2}$ was computed using the following equation:

(2) $$s_{B}^{2}=\frac{{\left(c_{t}\cdot \alpha -c\right)}^{2}}{\alpha \cdot \left(1-\alpha \right)}$$

where α is the cumulative sum of the probability up to the threshold *t*, c is the cumulative sum of the product of intensity levels and their probabilities up to the threshold *t*, and $c_{t}$ is the total mean of the entire histogram. Finally, an automatic threshold t is selected based on the maximum value obtained for the variance $s_{B}^{2}$, and this threshold is applied to the ECT image.

**Figure 10:**
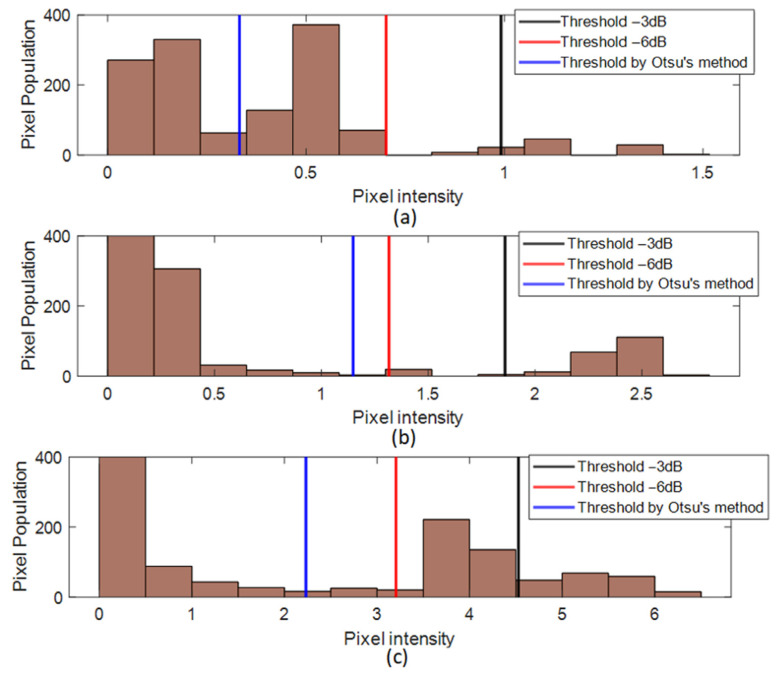
Histograms of pixel intensity distributions of the amplitude maps for the three delamination cases: (**a**) 5 mm delamination; (**b**) 20 mm delamination; (**c**) 50 mm delamination.

Figure 10 presents histograms of pixel intensity distributions for the amplitude maps obtained for the three delamination cases. The histogram maps also indicate the threshold values obtained for each thresholding approach. It should be noted that the histogram results presented in Figure 10 are obtained from the amplitude variation maps (Amp_erosion_). From Figure 10, it is possible to notice that the values obtained by the three different thresholding approaches are significantly different and that the application of these thresholding methods affect the estimated size of the delamination. In the case of using a fixed thresholding value of −3 dB, the histograms show that a part of the pixel intensities that contain information about the delamination is removed. In the case of using a thresholding value of −6 dB, the information about the delamination will be preserved in the histograms of pixel intensities. The use of the automatic thresholding method by applying the Otsu’s condition also preserves the information regarding delamination. However, for the case with a low amplitude variation, such as the 5 mm delamination, the method also retained significant background noise.

## 7. Delamination Imaging and Sizing

**Figure 11:**
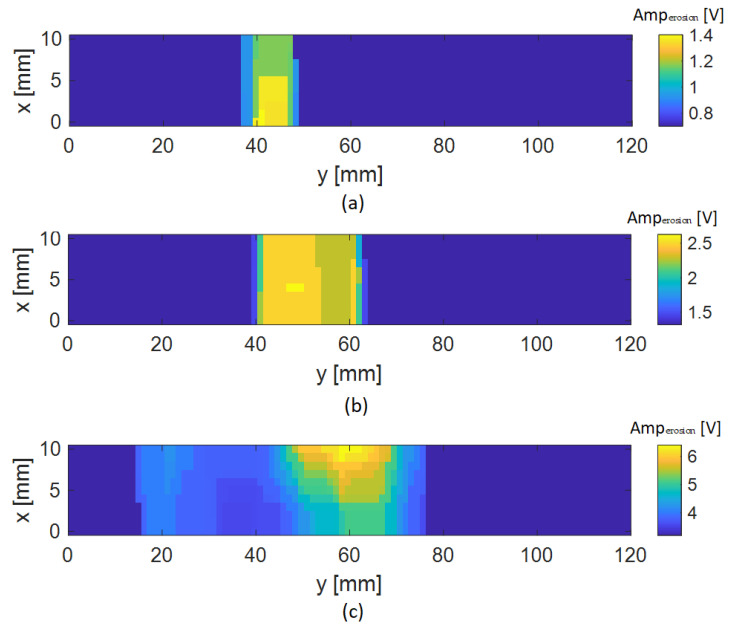
Resulting images were obtained after applying both thresholding at −6 dB and erosion for the three delamination cases: (**a**) 5 mm delamination; (**b**) 20 mm delamination; (**c**) 50 mm delamination.

An estimation of the length of each delamination was made considering both the erosion and thresholding methods or considering only the thresholding methods without using the erosion method. Table 1 summarizes the results obtained considering different scenarios. The results presented in Table 1 were obtained considering the mean of the delamination length estimated for 11 scans along the y-axis, crossing the defective region. The results show that the length of the delaminations is mostly overestimated without using erosion. Comparing the results of applying erosion before thresholding with those of applying thresholding before erosion indicates that performing erosion after thresholding results in a more accurate estimation of the delamination lengths. In the case where the delamination length is in the order of the width of the ECT probe, all the results remain overestimated. The best estimation of the length of the delamination was obtained by considering a thresholding value of −6 dB, which represents a threshold of 50% of the maximum amplitude variation of the image. The results of the images obtained for the best scenario are shown in Figure 11. It should be noted that the image processing techniques were implemented in grayscale images, but the colormap of the resulting images were converted back to the original colormap images for better visualization.

**Figure 12:**
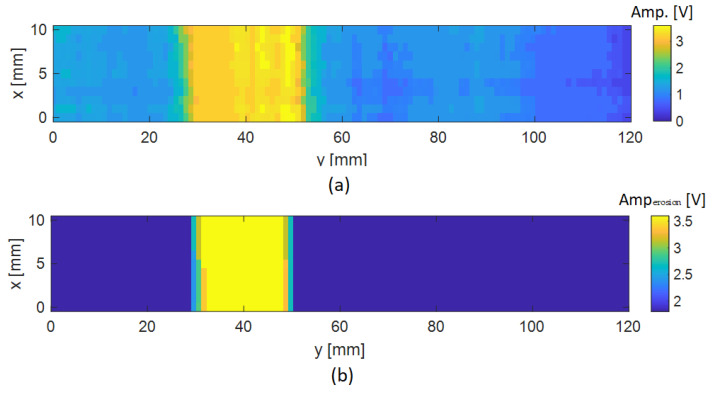
Results for the T2 sample containing a delamination depth of 0.14 mm with respect to the surface close to the ECT probe: (**a**) original C-scan; (**b**) after applying both thresholding at −6 dB and erosion process.

So far, the C-scan maps presented were obtained in the case where the delamination was located at the deepest layer of the composite with respect to the surface close to the ECT probe. To further evaluate the performance of the methodology, another C-scan was performed on the other side of the specimen, which provides a delamination depth of 0.14 mm with respect to the surface close to the ECT probe. In this case, Figure 12 shows the original C-scan and processed results for the CFRP plate referred to as T2. As expected, with the delamination close to the surface of the plate, the perturbed amplitude observed was greater than that noted in the case where the delamination was inspected at a deeper position. In this case, the estimated length of the delamination was approximately 20 mm, showing the robustness of the methodology implemented in the two cases.

## 8. Conclusions

The double ECT probe presented in this paper was able to detect three delaminations inspected in the CFRP plates using two different types of excitation configurations. The results of the ECT images obtained with the two different excitation configurations were initially compared and analyzed. The peak amplitude of the signal in the receiver element and the phase shift between the excitation and receiver were considered as damage-sensitive features. The ECT images obtained from the best configuration were used for imaging and sizing the delaminations. Comparing both damage-sensitive features, the amplitude maps showed a clearer image of the regions containing the delaminations. From the amplitude maps, the influence of the ECT probe’s dimensions on the selected feature was reduced by applying the erosion method. The resulting images provide a more accurate representation of the actual defect dimensions within the CFRP specimens. For delamination size estimation, −3 dB, −6 dB, and automatic thresholding using Otsu’s method were applied and compared. For the data obtained using this ECT probe, the results show that the use of a threshold of −3 dB removed part of the pixel intensities that contain information about the delaminations. The automatic thresholding Otsu’s method provided a better estimation of the length of the dimensions of the delamination; however, in the case of low amplitude variations in the ECT images, it also preserved background noise, affecting the estimation of the size of the delamination. The best results were obtained when the thresholding methods were employed before applying the erosion method. For the three delaminations inspected in the CFRP plates, the use of a threshold of −6 dB that represents 50% of the maximum amplitude variation yielded the best results. Regarding future work, this study can be extended by focusing the proposed methodologies on imaging and characterizing delaminations in other types of CFRP materials, such as woven composites, where eddy currents can exhibit more complex behavior. Additionally, the estimation of the location of the delamination depth can be an extension of the current study.

## Figures and Tables

**Table 1:** Summary of sizing delamination results with different approaches.

Approx. Length of the Delamination [mm]	Only Threshold			Erosion→Threshold			Threshold→Erosion		
	−3 dB	−6 dB	Otsu’s	−3 dB	−6 dB	Otsu’s	−3 dB	−6 dB	Otsu’s
5	11.00	16.18	16.18	7.00	9.73	67.27	5.91	9.73	9.73
20	22.18	24.45	24.45	18.00	19.73	20.00	17.64	19.73	19.73
50	20.09	54.36	57.64	17.27	51.18	54.45	13.91	49.64	53.00

## Data Availability

The data presented in this study are available on request from the corresponding author.
